# Tularemia induces different biochemical responses in BALB/c mice and common voles

**DOI:** 10.1186/1471-2334-9-101

**Published:** 2009-06-26

**Authors:** Hana Bandouchova, Jana Sedlackova, Miroslav Pohanka, Ladislav Novotny, Martin Hubalek, Frantisek Treml, Frantisek Vitula, Jiri Pikula

**Affiliations:** 1 Department of Veterinary Ecology and Environmental Protection, Faculty of Veterinary Hygiene and Ecology, University of Veterinary and Pharmaceutical Sciences, Brno, Czech Republic; 2 Centre of Advanced Studies, University of Defence, Hradec Kralove, Czech Republic; 3 Institute of Molecular Pathology, University of Defence, Hradec Kralove, Czech Republic; 4 Department of Infectious Diseases and Epizootiology, Faculty of Veterinary Medicine, University of Veterinary and Pharmaceutical Sciences, Brno, Czech Republic

## Abstract

**Background:**

Both BALB/c mice and common voles (*Microtus arvalis*) are considered highly susceptible to tularemia. However, the common vole is reported to harbour *Francisella tularensis *in European habitats as well as to survive longer with chronic shedding of the bacterium. The purpose of the present study was to compare the response of these two rodents to a wild *Francisella tularensis *subsp. *holarctica *strain infection.

**Methods:**

Rodents were evaluated for differences in the total antioxidant capacity derived from low-molecular-weight antioxidants, biochemistry including lipid metabolism, tissue bacterial burdens and histopathology following experimental intraperitoneal infection with 160 colony forming units (CFU) *pro toto*.

**Results:**

Bacterial burdens in common voles started to develop later post-exposure and amounted to lower levels than in BALB/c mice. Elevation of liver function enzymes was more pronounced in mice than common voles and there were marked differences in lipid metabolism in the course of tularemia in these two species. Hypertriglyceridemia and hypercholesterolemia developed in mice, while physiologically higher levels of triglycerides and cholesterol showed a decreasing tendency in common voles.

On the other hand, the total plasma antioxidant capacity gradually dropped to 81.5% in mice on day 5 post-infection, while it increased to 130% on day 6 post-infection in common voles. Significant correlations between tissue bacterial burdens and several biochemical parameters were found.

**Conclusion:**

As differences in lipid metabolism and the total antioxidant capacity of highly susceptible rodent species were demonstrated, the role of triglycerides, cholesterol and antioxidants in tularemic sepsis should be further investigated.

## Background

Tularemia, a zoonosis of veterinary and public health concern, is currently considered a re-emerging disease [1] and, due to the highly infectious nature of *Francisella tularensis*, it is also a potential biological weapon and a bio-terrorism agent [2]. Natural foci of tularemia may be characterized by long-term persistence under favourable environmental conditions [3] and the *F. tularensis *infection has been documented in a wide range of hosts [4,5] in which it manifests in multiple forms depending on the route of exposure. Ulceroglandular, glandular, oculoglandular, oropharyngeal, pneumonic, typhoidal and septic forms are the most frequent [6]. The inhalation route, in particular, can result in overwhelming sepsis associated with the highest rates of mortality in susceptible hosts [7]. Nevertheless, other routes of exposure also result in bacteraemia and dissemination of the bacteria to organs such as the liver, lungs, spleen and kidneys [8-10].

In sepsis, the widespread activation of cells responsive to bacteria or bacterial components generally results in the release of an array of inflammatory mediators, such as cytokines, chemokines, prostaglandins, lipid mediators, and reactive oxygen species [11]. Alterations in protein and lipid metabolism have been well documented in sepsis [12]. Reactive nitrogen species (RNS) and reactive oxygen species (ROS) are intermediates involved in the host defence against various intracellular pathogens including *F. tularensis*. Production of reactive molecular species is induced in macrophages when they are exposed to pro-inflammatory cytokines, including IFN-γ and TNF-β. After activation, macrophages are capable of arresting bacterial replication [13]. *F*. *tularensis *is exposed to ROS and RNS not only in macrophages but also in other cell types or extracellularly in vivo and both *F. tularensis tularensis *and *holarctica *subspecies are assumed to be virulent as they are armed with a variety of enzymes that can combat host ROS- and RNS-mediated killing mechanisms [14]. It has been demonstrated that the *F. tularensis *stress response required for its survival in diverse hostile environments is dependent on the MglA transcriptional regulator of genes contributing to virulence by encoding the *Francisella *pathogenicity island [15]. Some studies suggest that the *F. tularensis *infection confers an oxidative stress upon the target cells and that many of the host-defence mechanisms appear to be intended to counteract this stress [16]. Cells are equipped with defence mechanisms that provide protection via enzymatic activities or through low-molecular-weight antioxidants (LMWAs) acting as chemical scavengers and neutralizing reactive molecular species [17]. It is possible to measure the total antioxidant capacity (TAC) as a clinical marker of oxidative stress [18].

Rodents such as the laboratory mouse and common voles (*Microtus arvalis*) are considered highly susceptible to tularemia [19]. On the other hand, there are reports of chronic shedding tularemia nephritis in rodents [20], seropositive animals remaining culture-positive for *F. tularensis *[21] and chronic infection in species that are highly sensitive to tularemia [22,23].

These controversial findings demonstrate the need for further studies of the responses of various rodents to tularemia. The aim of the present study was, therefore, to use a wild *F. tularensis *strain and compare the development of tularemia in two species of rodents. One of them was the laboratory BALB/c mouse, representing an important model organism for experimental infection with *F. tularensis*, the other was the common vole (*Microtus arvalis*), which is reported to harbour *F. tularensis *with a prevalence as high as 3.9% during activation of tularemic foci in European habitats [24,25]. For comparative purposes we evaluated differences in the total antioxidant capacity derived from the low-molecular-weight antioxidants, biochemical responses including lipid metabolism and tissue bacterial burdens. The analyzed biochemical parameters are commonly available at every laboratory engaged with clinical biochemistry. However, to the best of our knowledge, detailed biochemical characteristics of tularemia-infected hosts have not yet been reported. The selection included parameters necessary for the evaluation of the general state of health, liver and kidney functions and energy metabolism.

## Methods

### 1) *Francisella tularensis *strain subtyping

A wild strain of *F. tularensis *isolated from a European brown hare specimen from South Moravia in 2004 was used for experimental infections in this study. The isolate was classified as *Francisella tularensis *subsp. *holarctica *using the proteomic procedure of *F. tularensis *subtyping that is described in greater detail elsewhere [26,27].

### 2) Preparation of *Francisella tularensis *for experimental infection

Experimental infections were performed using a suspension of *F. tularensis *cells washed down from a culture growing on blood agar supplemented with cystine using sterile physiological saline solution. No adjuvant was employed. After thorough mixing we measured the absorbance of the suspension at 605 nm using a spectrophotometer (Unicam Helios Gamma&Delta, Spectronic Unicam, United Kingdom) in order to determine the number of bacterial cells per unit volume according to McFarland's standard [28]. The number obtained was only approximate and was used to estimate the dilution necessary to achieve the dose required. The exact infectious dose was then determined by plating 10-fold serial dilutions and counting colony forming units (CFU) in the suspension administered to experimental animals. Colonies were counted after 72 h of incubation at 37°C.

### 3) Experimental animals

Two small mammalian species, i.e. the laboratory mouse (*Mus musculus*) and the common vole (*Microtus arvalis*), were used for the study. BALB/c mice were purchased from a commercial breeder and began the experiment at the age of 8 weeks with a body weight of 25 g. During early spring, parent common voles were live-trapped in the wild as over-wintered animals in a tularemia-free habitat and thus had no previous history of *F. tularensis *exposure. After checking their state of health (healthy appearance, excellent nutritional state, freedom from tularemia based on agglutination test) and sexing the captured specimens, pairs were formed and kept under laboratory conditions in boxes for rodents. All mortality cases in the breeding common voles were examined to exclude tularemia by culture and the mouse inoculation test. The offspring produced were used in the experiment, starting at the age of two months with a body weight of 14 to 21 g. Experimental animals were fed granules for rodents, a mixture of seeds, meadow grass and hay and were provided with drinking water *ad libitum*. Experiments were performed in compliance with laws for the protection of animals against cruelty and were approved by the Ethical Committee of the University of Veterinary and Pharmaceutical Sciences Brno, Czech Republic.

### 4) Biochemistry

Plasma chemistry profiles of laboratory mice and common voles were studied following intraperitoneal infection with 160 CFU *pro toto*. Of a total of 70 specimens of each species used in this experiment to evaluate the development of biochemical parameters in the course of tularemia, ten animals served as healthy controls and ten individuals were sampled every day post-infection. Blood was collected by cardiac puncture using a 2 ml heparinized syringe, centrifuged immediately, and plasma was removed and frozen (-20°C). Because of differences in survival of the studied rodents, it was only possible to obtain sufficient numbers of samples for 5 and 6 days in BALB/c mice and common voles, respectively. Within a few days, plasma was analysed using an automated analyzer (SPOTCHEM™ EZ SP-4430, ARKRAY, Japan) for creatinine (CREA; μmol/l), urea (UREA; mmol/l), aspartate aminotransferase (AST; μkat/l), alkaline phosphatase (ALP; μkat/l), alanine aminotransferase (ALT; μkat/l), lactate dehydrogenase (LD; μkat/l), creatine kinase (CK; μkat/l), total cholesterol (T-Chol; mmol/l), triglycerides (TG; mmol/l), glucose (GLU; mmol/l) and uric acid (UA; mmol/l).

### 5) Total antioxidant capacity

Cyclic voltammetry was used for estimation of the total antioxidant capacity derived from the plasma low-molecular-weight antioxidants (LMWA). The measured anodic current is proportional to the concentration of LMWA in the plasma sample. The principle of the assay employed in this study is described elsewhere [17,18]. Measurements were performed using the EmStat device (PalmSens, Houten, Netherlands) equipped with platinum working (1 mm diammeter), platinum auxiliary and silver/silver chloride electrodes screen-printed on a ceramic support (PalmSens). Electrodes were overlaid with 20 μl of plasma, and voltammetric curves were measured with a scanning rate of 100 mV/s. Data processing and device control were realized by the PSLite 1.7.3 software (PalmSens, Houten, Netherlands). The antioxidant capacity was estimated by means of the anodic current according to references [18,29]. Prior to measurements of experimental plasma samples the sensitivity of the device was tested and the cyclic voltammetry was calibrated by means of plasma spiking with ascorbate and cysteine. Cysteine was used as a representative of free thiol-bearing molecules that are oxidizable to the dithio form. Ascorbate was selected as another molecule participating as an antioxidant in the body.

### 6) Quantification of *Francisella tularensis *in tissues

Tissue bacterial burdens in organs of laboratory mice and common voles were studied following intraperitoneal infection with 160 CFU *pro toto*. After blood collection by cardiac puncture ten specimens of each species were killed by decapitation at 2, 3, 4, 5, and 6 days post-exposure. Cadavers were necropsied in order to examine gross pathological findings and collect organs aseptically (liver, kidney). To enumerate viable bacterial cells in 1 g of individual organs, samples were cut into small pieces and then homogenized using a homogenizer. After that, three dilutions were made in a 10-fold manner using sterile physiological saline. Taking 0.1 ml of each dilution, samples were plated and inspected for growth of colonies after 72 h of incubation at 37°C. Data on numbers of CFU in three dilutions from each organ and blood were then averaged and log_10_-transformed to obtain bacterial burdens per 1 g of tissue or 1 ml of blood.

### 7) Statistical analysis

Statistica for Windows 7.0 (StatSoft, Tulsa, OK, USA) was employed to evaluate differences among groups using the Tukey multiple comparison test. Values of p < 0.05 were considered statistically significant for all tests. Spearman rank order correlation analysis between tissue bacterial burdens and plasma chemistry profiles as well as the total antioxidant capacity was also employed.

## Results

### Biochemistry

**Figure 1:**
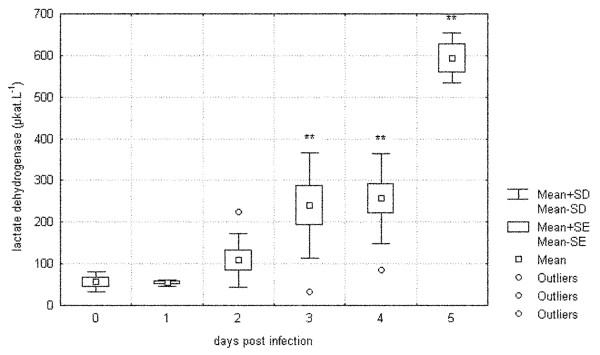
**Development of lactate dehydrogenase (LD) in the course of tularemia in BALB/c mice**. 0 = control group, 1 to 5 = groups of animals sampled on days 1 to 5 post infection, n = 10 in each group, * = p < 0.05, ** = p < 0.01 when compared against control group.

Tables 1, 2, 3 and 4 contain the plasma chemistry profiles and their development in the course of tularemia following intraperitoneal infection with 160 *F. tularensis *CFU *pro toto *in laboratory mice and common voles. Tularemia caused a 10-fold elevation of aspartate aminotransferase (AST) and lactate dehydrogenase (LD) in BALB/c mice. Figure 1 demonstrates that there was almost exponential growth of LD in mice. Alanine aminotransferase (ALT) activities also rose in mice by about 17-fold, whereas alkaline phosphatase (ALP) remained unchanged. Creatinine (CREA) increased on the day after exposure, while it showed a decreasing tendency toward terminal stages of infection. Creatine kinase (CK) changes were within clinically insignificant ranges. UREA rose only slightly and triglycerides (TG) and total cholesterol (T-Chol) in mice increased in the course of tularemia. With regards common voles, there were less pronounced changes in ALT and AST increased three-fold. On the other hand, ALP varied more than in mice, while LD activity had increased by only 5.5-fold in common voles on day 3 post-infection. As in BALB/c mice, CREA increased on the day after exposure in common voles. No clinically relevant changes in UREA and CK were noted in common voles. In contrast to BALB/c mice, initially high levels of TG and T-CHOL declined in the course of tularemia in common voles. Glucose (GLU) levels were significantly decreased right from day 1 post-infection both in mice and common voles. Uric acid (UA) levels were significantly increased on day 2 and day 1 post-infection in mice and common voles, respectively.

**Table 1:** Development of ALT, AST and ALP in the course of tularemia.

Groups	ALT		AST		ALP	
	
	*M. musculus*	*M. arvalis*	*M. musculus*	*M. arvalis*	*M. musculus*	*M. arvalis*
0	0.61 ± 0.31	1.66 ± 1.15	3.51 ± 1.99	3.91 ± 1.71	2.52 ± 0.44	5.84 ± 1.90

1	0.70 ± 0.14	4.04 ± 0.99	2.07 ± 0.13	14.54 ± 11.68	2.78 ± 1.02	6.24 ± 3.74

2	0.84 ± 0.07	2.16 ± 0.77	3.24 ± 1.19	7.12 ± 3.24	1.77 ± 1.00	6.75 ± 1.02

3	8.19 ± 7.22	6.36 ± 6.06	20.79 ± 18.51*	12.35 ± 10.96	2.78 ± 1.26	21.07 ± 18.74

4	8.38 ± 7.02*	4.96 ± 4.86	19.32 ± 15.81**	9.31 ± 5.32	2.11 ± 1.28	5.52 ± 1.03

5	10.52 ± 3.13**	2.59 ± 0.58	36.93 ± 4.83**	8.30 ± 1.60	2.27 ± 0.71	10.98 ± 8.06

6		2.37 ± 0.03		14.88 ± 4.77		7.42 ± 1.86

ALT = alanine aminotransferase (μkat/l), AST = aspartate aminotransferase (μkat/l), ALP = alkaline phosphatase (μkat/l), *M. musculus *= BALB/c mice, *M. arvalis = Microtus arvalis*, 0 = control group, 1 to 6 = groups of animals sampled on days 1 to 6 post-infection, n = 10 in each group, * = p < 0.05, ** = p < 0.01 when compared against control group. Values represent mean ± SD.

**Table 2:** Development of CREA, UREA and CK in the course of tularemia.

Groups	CREA		UREA		CK	
	
	*M. musculus*	*M. arvalis*	*M. musculus*	*M. arvalis*	*M. musculus*	*M. arvalis*
0	47.50 ± 3.89	41.33 ± 12.89	7.65 ± 0.98	6.84 ± 1.75	12.57 ± 6.13	14.76 ± 10.33

1	71.25 ± 6.11**	66.50 ± 24.06**	9.30 ± 2.67	6.87 ± 1.35	10.10 ± 3.18	11.87 ± 7.23

2	40.71 ± 12.95	33.33 ± 8.26	6.34 ± 1.65	5.40 ± 2.04	10.48 ± 7.45	12.12 ± 6.37

3	30.57 ± 2.97**	49.62 ± 14.31	12.57 ± 6.38	7.15 ± 3.29	18.42 ± 16.54	14.98 ± 9.98

4	30.22 ± 3.19**	41.50 ± 10.47	9.71 ± 6.92	7.86 ± 7.75	18.23 ± 13.38	15.04 ± 6.11

5	41.66 ± 1.80	47.60 ± 13.80	12.73 ± 6.97	4.46 ± 1.27	18.85 ± 6.17	16.63 ± 6.21

6		42.50 ± 1.58		8.15 ± 0.58		11.74 ± 1.53

CREA = creatinine (μmol/l), UREA = urea (mmol/l), CK = creatine kinase (μkat/l), *M. musculus *= BALB/c mice, *M. arvalis = Microtus arvalis*, 0 = control group, 1 to 6 = groups of animals sampled on days 1 to 6 post-infection, n = 10 in each group, * = p < 0.05, ** = p < 0.01 when compared against control group. Values represent mean ± SD.

**Table 3:** Development of LD, T-Chol and TG in the course of tularemia.

Groups	LD		T-Chol		TG	
	
	*M. musculus*	*M. arvalis*	*M. musculus*	*M. arvalis*	*M. musculus*	*M. arvalis*
0	55.92 ± 23.81	18.27 ± 11.14	2.25 ± 0.84	2.44 ± 1.06	1.24 ± 0.16	2.61 ± 0.88

1	54.28 ± 7.33	50.93 ± 49.81	1.83 ± 0.46	1.98 ± 1.02	1.30 ± 0.48	1.84 ± 0.59

2	108.34 ± 64.63	49.24 ± 34.08	2.26 ± 0.28	1.51 ± 0.23	1.22 ± 0.64	1.39 ± 1.05

3	240.21 ± 125.64**	109.81 ± 96.38*	3.67 ± 1.37*	1.73 ± 0.67	3.34 ± 2.19	1.01 ± 0.69*

4	256.54 ± 108.61**	33.05 ± 18.02	4.60 ± 0.88**	1.60 ± 0.38	3.42 ± 1.92	0.43 ± 0.24**

5	593.76 ± 59.55**	44.79 ± 10.56	3.79 ± 0.52*	1.89 ± 0.38	5.99 ± 3.99**	0.86 ± 0.39**

6		49.80 ± 13.14		1.39 ± 0.13		0.64 ± 0.16**

LD = lactate dehydrogenase (μkat/l), T-Chol = total cholesterol (mmol/l), TG = triglycerides (mmol/l), *M. musculus *= BALB/c mice, *M. arvalis = Microtus arvalis*, 0 = control group, 1 to 6 = groups of animals sampled on days 1 to 6 post-infection, n = 10 in each group, * = p < 0.05, ** = p < 0.01 when compared against control group. Values represent mean ± SD.

**Table 4:** Development of GLU, UA and Ia in the course of tularemia.

Groups	GLU		UA		Ia	
	
	*M. musculus*	*M. arvalis*	*M. musculus*	*M. arvalis*	*M. musculus*	*M. arvalis*
0	13.56 ± 1.72	10.73 ± 3.90	91.20 ± 14.44	233.80 ± 91.60	1796 ± 136	1793 ± 76

1	6.55 ± 0.89**	2.00 ± 1.20**	169.00 ± 18.62	419.00 ± 3.28*	1807 ± 44	1811 ± 95

2	8.68 ± 1.95**	4.07 ± 2.06**	337.16 ± 23.63**	209.25 ± 151.38	1624 ± 132	1842 ± 56

3	6.20 ± 3.33**	4.82 ± 1.95**	178.85 ± 15.97	298.00 ± 172.15	1570 ± 101*	1965 ± 125*

4	4.52 ± 1.20**	4.06 ± 2.07**	128.00 ± 40.99	190.00 ± 40.81	1516 ± 95**	2111 ± 96**

5	3.35 ± 0.38**	2.52 ± 0.52**	69.00 ± 1.09	263.75 ± 121.00	1463 ± 79**	2217 ± 163**

6		2.45 ± 0.49**		125.50 ± 13.69		2324 ± 134**

Ia = anodic current (nA) at 600 mV, GLU = glucose (mmol/l), UA = uric acid (mmol/l), *M. musculus *= BALB/c mice, *M. arvalis = Microtus arvalis*, 0 = control group, 1 to 6 = groups of animals sampled on days 1 to 6 post-infection, n = 10 in each group, * = p < 0.05, ** = p < 0.01 when compared against control group. Values represent mean ± SD.

### Total antioxidant capacity

Table 4 clearly demonstrates that the total plasma antioxidant capacity derived from LMWA and measured using cyclic voltammetry (anodic current Ia) was almost the same in control BALB/c mice and common voles. As tularemia progressed, LMWA levels gradually dropped to 81.5% in mice on day 5 post-infection, whereas they increased to about 130% on day 6 post-infection in common voles.

### Quantification of *Francisella tularensis *in tissues

The development of tissue bacterial burden in the blood, liver and kidney of BALB/c mice and common voles following intraperitoneal infection with 160 *F. tularensis *CFU *pro toto *at 2, 3, 4, 5, and 6 days post-exposure is presented in Table 5. As shown, the highest *F. tularensis *burdens were in the blood in both species. As demonstrated, bacterial burdens in common voles started to develop later post-exposure and amounted to lower levels than in laboratory mice.

**Table 5:** Development of tissue bacterial burden in the blood, liver and kidney of BALB/c mice (*M. musculus*) and common voles (*M. arvalis*) following intraperitoneal infection with 160 *F. tularensis *CFU *pro toto *at 2, 3, 4, 5, and 6 days post-exposure.

Days	BLOOD		LIVER		KIDNEY	
	
	*M. musculus*	*M. arvalis*	*M. musculus*	*M. arvalis*	*M. musculus*	*M. arvalis*
2	3.43	0	3.04	0	0	0

3	5.04	3.69	5.25	3.33	2.39	0

4	8.32	5.10	7.98	4.33	3.60	3.17

5	8.29	6.00	8.06	5.67	4.08	4.06

6		7.02		6.29		4.38

Values were log_10_-transformed to obtain bacterial burdens of CFU per 1 g of tissue or 1 ml of blood and represent means (n = 10 specimens of mice and voles examined each day).

### Correlation between tissue bacterial burdens and plasma parameters

Statistically significant correlations were found in BALB/c mice between the blood bacterial burden and ALT (*n *= 10, *R *= 0.9, *p *= 0.03), LDH (*n *= 10, *R *= 0.9, *p *= 0.03), GLU (*n *= 10, *R *= -0.9, *p *= 0.03), Ia (*n *= 10, *R *= -0.9, *p *= 0.03), and the liver bacterial burden and T-CHOL (*n *= 10, *R *= 0.9, *p *= 0.03) and TG (*n *= 10, *R *= 0.9, *p *= 0.03), and the kidney bacterial burden and ALT (*n *= 10, *R *= 0.97, *p *= 0.004), LDH (*n *= 10, *R *= 0.97, *p *= 0.004), TG (*n *= 10, *R *= 0.97, *p *= 0.004), GLU (*n *= 10, *R *= -0.97, *p *= 0.004), Ia (*n *= 10, *R *= -0.97, *p *= 0.004). Statistically significant correlations in common voles were those between the blood bacterial burden and TG (*n *= 10, *R *= -0.81, *p *= 0.04), GLU (*n *= 10, *R *= -0.89, *p *= 0.01), Ia (*n *= 10, *R *= 0.98, *p *= 0.003), and the liver bacterial burden and TG (*n *= 10, *R *= -0.81, *p *= 0.04), GLU (*n *= 10, *R *= -0.89, *p *= 0.01), Ia (*n *= 10, *R *= 0.98, *p *= 0.003), and the kidney bacterial burden and GLU (*n *= 10, *R *= -0.94, *p *= 0.005), Ia (*n *= 10, *R *= 0.94, *p *= 0.005).

## Discussion

In this experimental study, the response of selected murine (BALB/c mouse) and microtine (common vole) rodent species to *F. tularensis *infection was evaluated using bacterial burden quantification, biochemistry to screen liver and kidney functions as well as lipid metabolism and the total antioxidant capacity derived from the plasma low-molecular-weight antioxidants. A wild strain of *F. tularensis *isolated from a European brown hare was used for this purpose. The dose of 160 CFU *pro toto *was selected because in a previous experiment it invariably led to infection and mortality with a median survival of 4.5 and 7 days in BALB/c mice and common voles, respectively [30]. Bacterial burdens in common voles started to develop later post-exposure and amounted to lower levels than in BALB/c mice.

There were interesting differences in the biochemical responses of BALB/c mice and common voles in the course of tularemia. Although some authors observed a lack of positive correlations between the degree of hepatic damage and liver function tests [9], results from this study (i.e., more pronounced elevations of ALT, AST and LD in mice compared to common voles) suggest the higher susceptibility of mice to *F. tularensis *infection. Interestingly, significant correlations between tissue bacterial burdens and some biochemical parameters were found. LD is found in skeletal and cardiac muscle, liver, kidney, bone and erythrocytes and elevations can be observed with disruption of any of these or in haemolysis. It is known that LD levels may be used to follow the progress of liver disease because they change more quickly. Distinction of the source of LD elevation depends on measuring CK originating mainly in skeletal and cardiac muscle. Elevated LD levels without concurrent elevation in CK in BALB/c mice and common voles are suggestive of hepatocellular damage. Leakage of LD from hepatocytes follows even functional alterations of mitochondria [31] so its elevation may reflect a low degree of hepatocellular damage. As shown in Tables 1 and 3, LD starts to rise earlier than ALT and AST in BALB/c mice and may thus be considered an important indicator of acute hepatocellular damage in tularemia [32]. Glucose levels significantly decreased in both rodent species right from day 1 post-infection, which is characteristic of severe sepsis as well as hepatocellular damage. Considering all the biochemical parameters evaluated in the present study, only the level of glucose showed significant changes within the first 2 days and remained significant during the following days.

The mild elevation of UREA in BALB/c mice was probably due to dehydration and catabolism in animals that ceased eating with the onset of clinical signs of tularemia, while elevations of CREA in mice and voles on the day after exposure were probably due to fever and the resulting dehydration. However, there were no signs of kidney failure in BALB/c mice and common voles.

Interestingly, there were marked differences in lipid metabolism in the course of tularemia in BALB/c mice and common voles. While levels of TG and T-CHOL steadily rose in BALB/c mice, they showed a decreasing tendency in common voles. Hypertriglyceridemia is known to occur as a response to bacterial endotoxin, and triglyceride-rich lipoproteins are thought to be agents of innate immunity in the host [33]. Elevation of TG during gram-negative sepsis results from decreased clearance of TG due to suppressed lipoprotein lipase activity and higher rates of TG synthesis [34]. The present study demonstrates that BALB/c mice responded to the infection by *F. tularensis *with cytokine-induced hyperlipoproteinemia [35], and the effects of pro-inflammatory cytokines [12] may be the reason for their short survival and high susceptibility to the infection by *F. tularensis*. On the other hand, natural levels of triglycerides in healthy common voles are higher than in BALB/c mice and it may be hypothesized that these may participate in conferring the higher innate resistance of common voles to tularemia compared with laboratory mice, because hypolipidemic animals are more sensitive to lipopolysaccharide-induced sepsis [11,36]. Data on the development of TG levels in common voles in the course of tularemia demonstrate TG hydrolysis and decreased cholesterol synthesis in this species [35].

In the present study, the total plasma antioxidant capacity gradually decreased in BALB/c mice, while, on the other hand, it increased in common voles in the course of tularemia. The rapid depletion of total antioxidants noted in BALB/c mice is further evidence of the higher susceptibility of this species to infection with *F. tularensis *compared with the common vole, which was able to respond to the developing infection by a gradual increase in antioxidant capacities. Generally, tissue bacterial burdens in common voles start to develop later post-exposure and amount to lower levels than in laboratory mice. The approximate tissue and blood burden of *F. tularensis *cells reached 10^4 ^CFU within two days in mice infected with the dose of 160 CFU, while the same infectious dose in common voles led to a burden of 10^4 ^within four days. The higher and more rapidly developing tissue and blood bacterial burdens of *F. tularensis *in mice result in higher levels of oxidative stress induced by the bacterium, which the host-defence mechanisms have to counteract as well as mobilize the required energy [37]. However, it seems that the antioxidant defence is quickly overcome by the infection in mice despite energy mobilization. A similar finding, i.e. that genes that lead to depletion of glutathione are upregulated following infection with *F. tularensis *LVS, has already been made *in vitro *using the mouse macrophage cell line J774 [37]. Another newly recognized mechanism that may lead to the drop in antioxidant glutathione is its utilization as a source of cysteine in the host cytosol [38]. On the other hand, the growing antioxidant capacity in the common vole may be responsible for its lower susceptibility to the infection by *F. tularensis*. The relation between antioxidant levels and disease progress is well known. For example, the antioxidant response was also recognized in cancer patients [39] using previously optimized measuring protocols [40] and in FIV infection in cats [41].

The advantage of cyclic voltammetry is that it determines the total antioxidant capacity based on the evaluation of a total reduction effect of individual LMWAs without their exact qualitative differentiation [18]. Calibration of the measuring system was performed using ascorbate and cysteine. However, the number of chemical antioxidants occurring in the body is much more extensive [42]. The limit of detection of isolated compounds is in the range of 1–10 μM. This range of sensitivity is sufficient for determining physiological concentrations of biologically relevant scavengers. It has been found that ascorbic and uric acids greatly contribute to the measured antioxidant capacity [43]. Therefore, UA levels were also measured separately using an automated biochemical analyzer in the present study. They were significantly increased on day 2 and day 1 post-infection in mice and common voles, respectively. It is, however, clear that in the present study UA levels do not correlate with the measured total antioxidant capacity.

There are some limitations of the study. First, experimental animals were inoculated intraperitoneally, i.e. via an unnatural route of infection. The decision to do so was made with the intention to induce sepsis as quickly as possible and examine biochemical responses in septic animals. Biochemical changes during other clinically more relevant routes of infection may be studied in future experiments. The intraperitoneal LD_50 _was approximately 1 and 38 colony forming units in BALB/c mice and common voles, respectively, in our previous study [30]. Common voles are thus slightly less susceptible to the *F. tularensis *subsp.*holarctica *infection. However, the difference in susceptibility is not so marked to explain the observed differences in biochemistry responses. Moreover, experimental animals were injected the dose many times exceeding the LD_50 _and some biochemical changes (metabolism of lipids, in particular) showed a reverse trend when comparing BALB/c mice and common voles. Second, only the BALB/c mouse strain was used in the current study. It is therefore unclear if the observed differences in bacterial burdens and biochemistry responses between BALB/c mice and common voles truly reflected the differences between the two rodent species or simply the differences between a given mouse strain and common voles. Responses of wild-type mice would be valuable for species comparisons.

## Conclusion

Differences in lipid metabolism and the total antioxidant capacity in two highly susceptible rodent species were demonstrated, and the role of triglycerides, cholesterol and antioxidants in tularemic sepsis should be further investigated. Evaluation of antioxidants may be used as a biomarker of disease progress. Changes in both the enzymatic and non-enzymatic antioxidant components might also be evaluated in future experiments to enhance our knowledge on the pathogenesis of this complex zoonotic disease.

## Competing interests

The authors declare that they have no competing interests.

## Authors' contributions

HB carried out the whole experiment, performed data analyses and took part in preparing the manuscript. JP planned the experiment, performed statistical analyses and participated in preparing the manuscript. JS prepared experimental animals, assisted in planning the experimental design and biochemical evaluations. MP analysed plasma samples using cyclic voltammetry. LN performed the histopathological examinations. MH subtyped the *Francisella tularensis *strain. FT and FV cultured *Francisella tularensis*, prepared the strain for experimental infection and quantified the bacterium in tissues. They also took part in preparing the manuscript. All authors contributed to the study design, the preparation of the manuscript and also read and approved the final manuscript.

## Pre-publication history

The pre-publication history for this paper can be accessed here:

http://www.biomedcentral.com/1471-2334/9/101/prepub
